# Next-generation study databases require FAIR, EHR-integrated, and scalable Electronic Data Capture for medical documentation and decision support

**DOI:** 10.1038/s41746-023-00994-6

**Published:** 2024-01-12

**Authors:** Martin Dugas, Max Blumenstock, Tobias Dittrich, Urs Eisenmann, Stephan Christoph Feder, Fleur Fritz-Kebede, Lucy J. Kessler, Maximilian Klass, Petra Knaup, Christoph U. Lehmann, Angela Merzweiler, Christian Niklas, Thomas M. Pausch, Nelly Zental, Matthias Ganzinger

**Affiliations:** 1 Institute of Medical Informatics, Heidelberg University Hospital, Heidelberg, Germany; 2 Department of Hematology, Oncology and Rheumatology, Heidelberg University Hospital, Heidelberg, Germany; 3 Department of General Internal Medicine and Psychosomatics, Heidelberg University Hospital, Heidelberg, Germany; 4 Department of Ophthalmology, Heidelberg University Hospital, Heidelberg, Germany; 5 Clinical Informatics Center, University of Texas Southwestern Medical Center, Dallas, TX USA; 6 Department of General, Visceral, and Transplantation Surgery, Heidelberg University Hospital, Heidelberg, Germany; 7 Department of Anesthesiology, Heidelberg University Hospital, Heidelberg, Germany

**Keywords:** Clinical trial design, Research data, Information technology, Drug development

## Abstract

Structured patient data play a key role in all types of clinical research. They are often collected in study databases for research purposes. In order to describe characteristics of a next-generation study database and assess the feasibility of its implementation a proof-of-concept study in a German university hospital was performed. Key characteristics identified include FAIR access to electronic case report forms (eCRF), regulatory compliant Electronic Data Capture (EDC), an EDC with electronic health record (EHR) integration, scalable EDC for medical documentation, patient generated data, and clinical decision support. In a local case study, we then successfully implemented a next-generation study database for 19 EDC systems (*n* = 2217 patients) that linked to i.s.h.med (Oracle Cerner) with the local EDC system called OpenEDC. Desiderata of next-generation study databases for patient data were identified from ongoing local clinical study projects in 11 clinical departments at Heidelberg University Hospital, Germany, a major tertiary referral hospital. We compiled and analyzed feature and functionality requests submitted to the OpenEDC team between May 2021 and July 2023. Next-generation study databases are technically and clinically feasible. Further research is needed to evaluate if our approach is feasible in a multi-center setting as well.

## Introduction

Structured patient data play a key role in all types of clinical research. These data have a well-defined schema or data model with data elements that have a defined meaning and format. Structured patient data are a key component in nearly every clinical study database and are valuable for research purposes, as data points for cross-patient analysis can be easily accessed and analyzed. High-volume data (imaging, sensor, genomic) for example need to be linked to structured patient attributes (e.g., presence of disease, age, gender, etc.) for data analysis to identify features which are associated with good or poor outcome of a disease in a population. Artificial intelligence (AI) algorithms for the interpretation of medical images or sensor data also require clinical diagnoses or findings (e.g., pneumothorax) for training and validation purposes.

The type of research that can be performed on structured data is significantly more powerful than on unstructured data. For example, when evidence for the efficacy of a new medical intervention needs to be established, the strongest tool is a randomized controlled trial (RCT)^1^. In RCTs, data are collected prospectively and they must be of high quality. While RCTs create a standardized environment for the intervention, other conditions in the patient’s experience may be very diverse. These deviations might affect efficacy, thus studies on Real-World Data (RWD) and Real World Evidence (RWE) play an increasingly important role in assessing medical products and interventions^2^. Potential sources for RWD include electronic health records (EHR) and insurance claims data^3^.

**Fig. 1:**
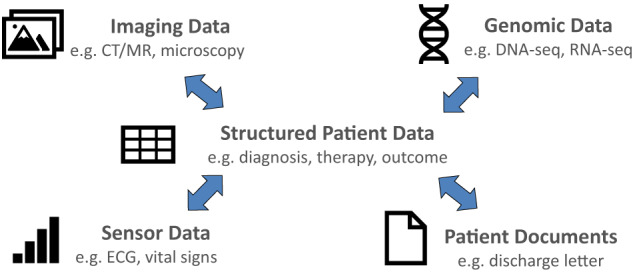
Categories of patient data. Structured patient data play a key role in nearly every clinical study database.

Patient data cover a wide range of categories, as illustrated in Fig. 1. Structured patient data can be represented in a tabular format and consist of many different variables such as diagnosis code, therapeutic procedure, vital signs, or outcome data like response to treatment or survival time. Due to the variety of medical terminology, variables may contain thousands of different attributes. The attribute scope is best demonstrated by the systematized nomenclature of medicine (SNOMED), which contains more than 300,000 non-synonymous concepts^4^. In principle, each SNOMED concept can be an attribute of a structured patient data variable. Obviously, this estimate is conservative because many medical terms require post-coordination of SNOMED codes.

Imaging data constitute another major category of patient data. Data are generated from imaging modalities like computer tomography or microscopy and are characterized by large data volumes per patient. Likewise, sensor data, such as electrocardiograms, have been traditionally collected in an intensive care unit or perioperative setting. Recently, wearable devices like smart watches became another source of sensor data. Genomic data are mostly generated by sequencing of human material. Resulting data sets can be very large, for example, when a patient’s whole genome is analyzed.

Finally, unstructured data in patient documents are a frequently used data category. Today, a large portion of clinical information in EHR systems is still stored in free text documents such as clinical notes and discharge letters^5^. Unstructured data do not have a predefined data model. Such data still contain valuable medical information, but this is not directly accessible for analysis. Data first have to be extracted into a structured data model, either manually or by means of natural language understanding (NLU) algorithms^6^.

**Fig. 2:**
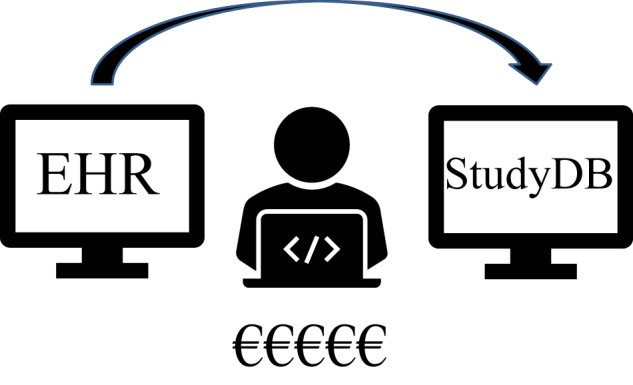
Manual data transfer from EHRs into study databases is currently the most frequently used method of data collection in interventional clinical trials. It is a slow, error-prone, and expensive process.

Due to their importance for clinical research, structured patient data are captured within validated study databases. In contrast, most commercial EHR systems are currently not designed for clinical research and the majority of content is comprised of unstructured data^5,7^. Therefore, for the purpose of research, many data points are extracted by manual chart review from EHR systems and entered into study databases via electronic case report forms (eCRFs) (cf. Fig. 2), a laborious and very costly process making clinical research very expensive^8^. For example, Pronker et al. estimated a cost of €200,000 for the correction of erroneous data in three trials^9^.

Development of new drugs is extremely costly (DiMasi et al. estimate capitalized cost for research and development of $2,870 million per approved drug^10^) and therefore is generally the sole domain of large pharmaceutical companies. A significant portion of these development costs relate to clinical studies, in particular prospective interventional trials. Developing an improved study database design with lower cost per valid data point could improve feasibility and delivery time of clinical studies. Further, it could foster investigator-initiated trials (IITs), which are driven primarily by medical needs and not by economic interests.

The objectives of this manuscript are to answer the following research questions:

- What general characteristics are required for study databases to improve efficiency and effectiveness of the data collection process?
- Is it feasible to implement and operate such an improved database in today’s setting of a university hospital?

We performed our study and proof of concept implementation at the Heidelberg University Hospital, a major tertiary referral hospital in Germany with approximately 80,000 inpatient admissions and more than one million outpatient visits per year. In this context, we elicited requirements for a next-generation study database as outlined in our first objective. To test these requirements for validity and feasibility, we implemented a prototype at the Heidelberg University Hospital for 11 clinical departments.

## Results

Corresponding to the dual objectives of this study, two result sections are presented. First, the requirements relevant for a next-generation study database are described. They were derived from users’ requests and cover a wide variety of aspects such as data comparability, EHR integration, and patient generated data. This section is followed by the description of a prototype software implementation at Heidelberg University Hospital based on these requirements.

### Requirements

New study results must be compared to and interpreted in the context of prior study results. Therefore, researchers should consider reuse of data structures from prior studies of the same disease at the design stage of a new study database. Following the *FAIR* data principles of findability, accessibility, interoperability, and reusability^11^ can foster access to prior studies’ structural metadata (e.g., eCRFs). In conjunction with *semantic annotation* of data elements (to provide clear definitions for each data element), FAIR access to metadata can foster data compatibility between new studies and prior research.

If clinical research is expected to be practice changing (e.g., new diagnostic or therapeutic approaches), approval from regulatory authorities is required. This means that study databases must *conform to regulatory data standards*. A key requirement in this context is traceability of data point origins (audit trail) to establish trust in the data, which is provided by Clinical Data Interchange Standards Consortium (CDISC, https://www.cdisc.org) conform systems. Many other commonly used data collection tools (e.g., Microsoft Excel, IBM SPSS Statistics, or similar software) do not meet the demand for an audit trail during data capture.

Ideally, EHR data should be well-structured, of high quality, and suitable for clinical research. If that is the case, electronic data capture could be done through *EHR-integration*, potentially bi-directionally. Import of EHR data into the electronic data capture (EDC) should be possible (e.g., extraction of laboratory results) and export should be feasible as well (e.g., a clinical note derived from a structured EDC of a patient visit into the EHR). While EHR data include all categories of clinical data (see also Fig. 1), research often mandates more data points than generated in routine clinical care (including additional diagnostic procedures or more visits for a detailed patient follow-up). Therefore, extraction, transformation, and loading (ETL) of data from an EHR system can only contribute a subset of data for a study database, requiring a combination of EHR and EDC data.

EDC systems need to be disease-specific and adaptable to a specific study setting (e.g., a clinical study about acute myeloid leukemia). With more than 10,000 coarsely-grained diagnoses in the International statistical classification of diseases and related health problems (ICD)^12^, *scalable software development* methods for disease-specific systems are needed to facilitate implementation of study databases. Model-driven software development is an established method and allows generating software from a model description in contrast to error-prone and laborious manual software programming.

To improve outcome research, clinical study databases should not only contain comprehensive, high data quality that cover the full scope of *medical documentation* (e.g., inpatient and outpatient visits capturing all data generated by healthcare professionals) but also include *patient generated data*, such as patient reported outcome measures (PROMs)^13^. PROMs would permit extended plausibility checks, identification of biases in the data, and detailed assessment of diagnostic and therapeutic effects.

Study databases as well as *clinical decision support* (CDS) used in patient care depend on high-quality structured data. Integrated systems for clinical research and routine care should avoid redundant data capture. Given the general resource constraints of clinical research and care, every data point should be documented only once and be re-used when necessary. CDS could be used to improve adherence to medical guidelines, foster patient safety, and at the same time support research study workflows.

Based on the described desiderata for study databases, a *next-generation study database* should have the following characteristics:

1. Backward Compatibility through FAIR access to eCRFs (e.g., structural metadata) with semantic annotation
2. Conformity with regulatory data standards and audit trail capability
3. Integration of EDC and EHR
4. Scalable EDC
5. Integration of clinical team documentation with the EDC system.
6. Collection of patient-generated data (e.g., PROMs)
7. Clinical decision support leveraging integrated data from EHR and EDC

### Prototype software implementation

A key requirement for the prototype implementation is a regulatory compliant and scalable EDC software. For our case study, we used OpenEDC^14^ as the EDC system based on CDISC’s operational data model (ODM)^15^. OpenEDC can import eCRFs in CDISC ODM format. In addition, the portal of medical data models^16^ (MDM-Portal, https://medical-data-models.org/) provides over 25,000 CRFs with semantic annotations, which can be imported directly into OpenEDC and adapted to study-specific documentation needs. Reuse of CRFs from MDM portal makes EDC development more scalable by allowing new studies to reuse elements, which reduces workload for medical design, programming, and testing. Since OpenEDC is based on ODM, the semantic annotations of the forms’ definitions are also included in the final data file alongside stored questionnaire responses.

**Fig. 3:**
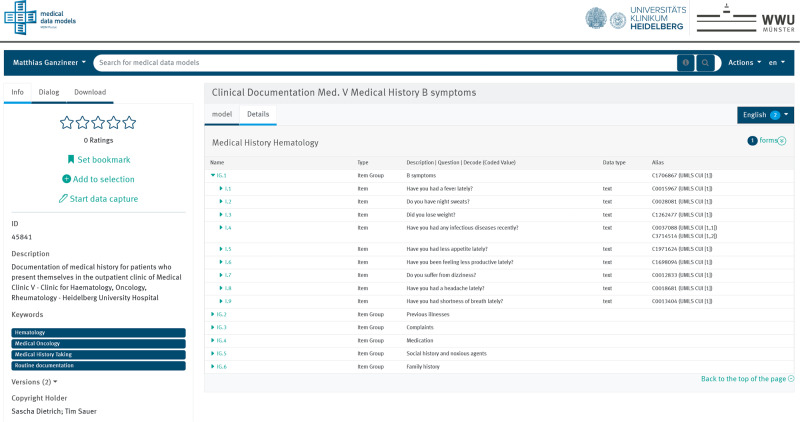
Data model of the questionnaire with semantic annotation (UMLS codes for data elements). Available from MDM-Portal (https://medical-data-models.org/45841).

In order to provide FAIR access to eCRFs with semantic annotation the case study, we uploaded the eCRFs to collect the medical history to the MDM portal (Fig. 3). All data elements were annotated with Unified Medical Language System (UMLS)^17^ codes by physicians to provide language-independent semantic annotation.

**Fig. 4:**
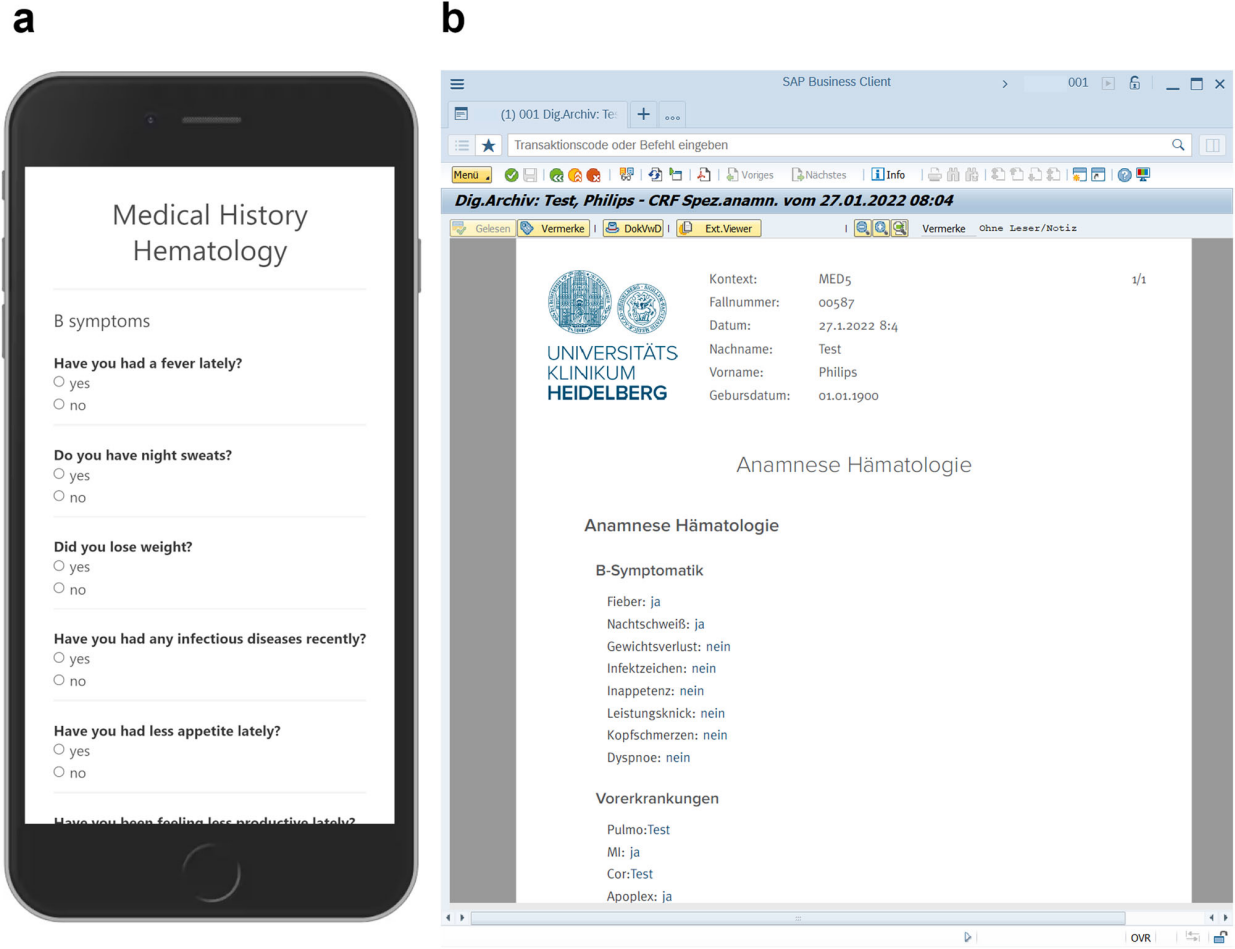
Sample questionnaire for Digital Medical History. Questions address fever, night sweats, weight loss, infectious diseases, lack of appetite, general performance, etc. **a** Representation of questionnaire on tablet. **b** Document in EHR generated from tablet data (in German). Both artifacts were generated from the data model in the previous figure. Note: No actual patient data are presented here.

Figure 4 presents an example for integration of EDC and EHR data: After patient questionnaires were collected with an iPad, a PDF report was transferred into the EHR system in addition to data storage in the EDC database.

In addition, EDC was integrated into the hospital’s clinical EHR workstations: In the EHR’s patient list, we implemented a web link to the EDC system for the clinical team to document in the EDC (see also Supplementary Fig. 1). This link contains the patient record number, current clinical user identification, and an encrypted hash. Selecting the link opens the EDC system while maintaining the context of the currently selected patient from the EHR list and the current clinical user. The encrypted hash provides a secure mechanism for logging into the EDC systems without requiring additional password entry.

**Fig. 5:**
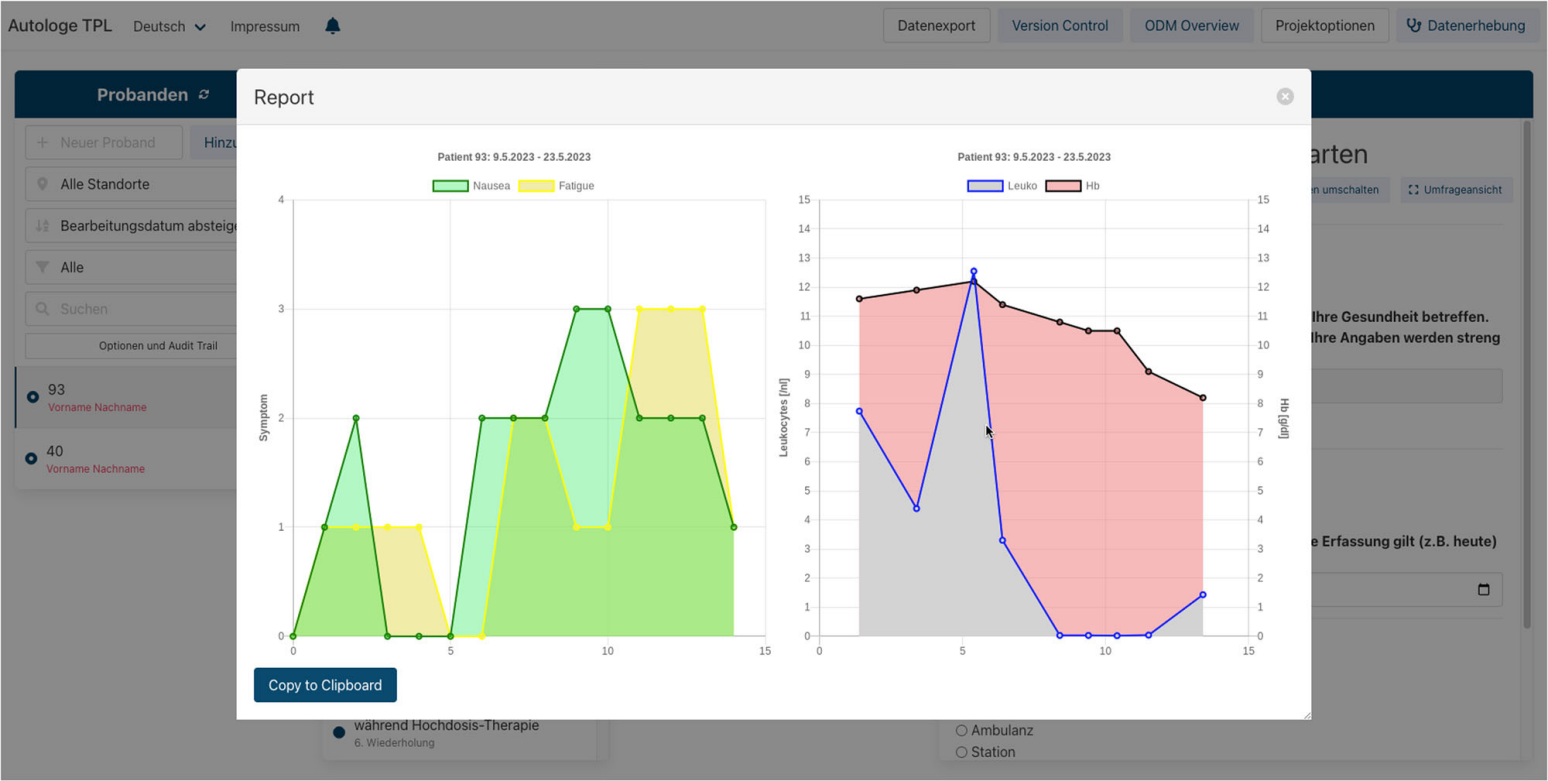
Patient summary in the EHR generated from EDC system. Using the report feature in the EDC system, a summary of PROM data (nausea, fatigue) and laboratory data (hemoglobin, leukocytes) is generated and displayed as a chart.

Patients in internal medicine completed identical questionnaires at follow-up visits as often as 15 times per patient. We developed a prototype for a patient summary as an example for clinical decision support. This overview page presents the course of symptoms over time (available from EDC). These findings are integrated with selected laboratory parameters over time (available via ETL from the EHR system) to allow CDS functionality (see Fig. 5).

## Discussion

Today, EDC and EHR data are usually not integrated. However, data collected in clinical care and in research at university hospitals are closely related. Informed by 19 EDC projects from Heidelberg University Hospital, we collected, consolidated, analyzed, and prioritized feature requests for improved study databases. We identified seven characteristics of *next-generation study databases* and performed a local proof of concept study to assess technical and clinical feasibility of this approach. We engaged 11 clinical departments with this study making it more likely that our findings would be generalizable and applicable to other clinical settings. Overall, we demonstrated that next-generation study databases are feasible in the current clinical setting.

We showed the feasibility of *FAIR access to eCRFs* by uploading medical history forms to the MDM portal. MDM and OpenEDC provide semantic annotation of data elements with UMLS codes thus meeting this criterion. Despite several public demands for FAIR principles in clinical research^11,18^, at present a vast portion of clinical studies does not meet FAIR principles. At the time of manuscript preparation, clinicaltrials.gov (https://clinicaltrials.gov) listed more than 456,000 studies^19^, turning this requirement into a large-scale challenge. The feasibility of data comparison with prior research is greatly facilitated by compatible and congruent data structures in previous and new study databases. There is a paucity of available, sharable CRFs (structural metadata) and only eligibility criteria are published on clinicaltrials.gov. For some studies, subject-level data may be requested, but the usefulness of the data is limited due to the absence of related CRFs explaining the data collection. A very good exception from such “unFAIR” studies is the database of Genotypes and Phenotypes (dbGaP) from NCBI^20^, which provides descriptive and structural metadata for over 2,000 studies. On request, patient-level data are also available. Recently, metadata from dbGaP with semantic annotations were made available in the MDM portal for 585 studies (as of August 9, 2023). Re-use of well-proven CRFs can improve comparability of study data. Semantic annotation of data elements facilitates data integration with prior studies, as it supports mapping of data elements between different data sources by matching semantic codes. Interpretation of new study data must include comparisons with prior study results. To facilitate data integration between different studies, corresponding data elements need to be mapped, which is facilitated by semantic annotation of data elements (e.g., Logical Observation Identifier Names and Codes, LOINC, for laboratory data^21^).

Study databases must comply with regulatory standards to enable data submissions from prospective and interventional studies to regulatory agencies such as the European Medicines Agency (EMA), United States Food and Drug Administration (FDA), Pharmaceuticals and Medical Devices Agency (PMDA) in Japan, or National Medical Products Administration (NMPA) in China. Therefore, the use of data formats endorsed by these agencies is preferable to minimize efforts for data preparation during the submission process. Regulatory agencies have agreed on standards issued by the Clinical Data Standards Interchange Consortium (CDISC, https://www.cdisc.org). While FDA^22^, PMDA^23^, and NMPA^24^ have endorsed CDISC standards or even require those for data submission, EMA is preparing to adopt these standards^25^. For EDC systems, CDISC ODM is especially suitable, since it was designed as an exchange format for clinical metadata and data^15^. CDISC ODM also supports an audit trail, which is another key feature of regulatory-compliant data collection (traceability of data point origin).

At present, the EDC remains separate from the EHR. Manual review of EHR charts is performed for source data verification. *EHR and EDC data integration* can have several benefits: EDC data can be used to enrich EHR data and to avoid redundant data entry, when for example patient questionnaires are transferred from the EDC into the EHR systems. Our proof-of-concept study demonstrated that this is feasible with standard functionalities of the EHR legacy systems (Fig. 4). Calling EDC data from within the EHR can simplify and enrich medical documentation for the clinical team working primarily with the EHR system. We demonstrated that access to EDC data from the EHR is feasible, but—from our experience—is technically more demanding as it requires a single-sign-on functionality for the EHR and the EDC and extended configuration capabilities to maintain patient context. However, single-sign-on is important for clinical acceptance.

Further, the validity of EDC data will be improved, if they are also used directly in routine care. If data quality criteria for EDC are met, data transfer from the EHR into the EDC is possible (e.g., laboratory data). The Observational Medical Outcomes Partnership (OMOP) successfully established retrospective analysis of EHR data^26^. For new diagnostic and therapeutic procedures, we need prospective clinical studies. The strong regulatory requirements for interventional trials require validation procedures with a focus on data quality. Further research is needed to assess automated data transfer from the EHR into the EDC systems for interventional trials.

EDC *software development* is complex, because it must reflect the complexity of clinical medicine with a high degree of verisimilitude. More than 10,000 diagnoses in ICD-10^12^ primarily correspond to billing requirements. For each diagnosis used, specific data elements are needed in an EDC system. More finely grained terminologies such as SNOMED address more specific aspects of clinical research. As shown on clinicaltrials.gov, hundreds of thousands of clinical studies were conducted and each may have a different data structure to address the study’s protocol requirements. Typically, each study captures hundreds of disease-specific data elements making the design, development, maintenance, and quality control of the EDC resource intense. Thus, efficient implementation of study databases is a key success factor for clinical research.

In EDC development, the current state-of-the-art is manual development of EDC systems’ data field by data field. Underutilized, the re-use of eCRFs from prior studies and (semi-)automatic generation of study databases instead of manual programming can make EDCs more scalable. However, the large variety of EDC systems (more than 90 systems according to G2, https://www.g2.com/categories/electronic-data-capture-edc) and metadata formats for eCRF design do not support the sharing of eCRFs among EDC systems. It would be highly desirable for researchers if all EDC systems would support the importing of metadata based on the FDA data standards (at present CDISC ODM) to foster re-use of CRFs. In our case study, we used OpenEDC^14^, because it is an open-source EDC system, which can import eCRFs in CDISC ODM format. Additionally, OpenEDC is integrated into the MDM portal and with only one click (“start data capture”) in the MDM portal, an OpenEDC database is created on the local computer allowing instant data collection.

*Medical documentation* by healthcare professionals in the course of delivering care is a key source for data for EDC systems. While large semantic overlaps between routine documentation and study documentation can be found, study documentation is much more detailed and structured than EHR data. Therefore, it should be feasible to transfer summaries of clinically relevant aspects from the EDC data into the EHR. Re-use of patient data from other systems (e.g., laboratory information systems) also avoids error-prone redundant data entry. In our proof of concept study, we demonstrated the technical feasibility of a patient summary with EHR integration and ETL of laboratory data. As a long-term perspective, a comprehensive EHR with the full patient journey would also provide new opportunities for medical research.

*Patient generated data* such as the medical history or patient reported outcomes (PROs) are important for routine care and research. Our proof of concept study demonstrated that non-redundant collection of these data in the EDC and the EHR systems is technically and clinically feasible using a standardized EHR interface for PDF-transfer with an HL7-based communication server (HL7-MDM-interface). Because the patients enter the data, data collection was feasible without additional clinical personnel in a resource-limited environment. A frequent non-technical problem when collecting PRO are license restrictions for PRO forms usage. Paying a license fee for a PRO form for each patient generates a large financial and administrative overhead barring large-scale implementation. Development of free PRO instruments should be encouraged through public initiatives.

Currently, EDC data are collected separately from clinical data and therefore cannot contribute to *clinical decision support*. CDS may improve the adherence to clinical guidelines and contribute to more accurate diagnoses^27^, patient safety, and treatment quality. CDS depends on (near) real-time, high quality clinical data. Thus, an EHR integrated EDC could contribute data required for CDS. Examples include calculations of disease scores or proposed diagnostic or therapeutic procedures for a patient^28^. However, at least in the European Union presenting more than basic data points makes CDS a medical device, which must be developed and placed on the market according to the medical device regulation (MDR)^29^. The resulting workload for software manufacturers and software operations managers may be prohibitive. Further research is required to determine if CDS based on EDC data can provide additional clinical benefits justifying the additional MDR efforts.

Many publications discuss the interoperability of medical information systems based on the FHIR standard allowing the re-use of EHR data in retrospective studies. The Observational Health Data Sciences and Informatics (OHDSI)^30^ community has very successfully demonstrated the power of open-source systems for large-scale health data analytics in observational settings. We propose to focus on regulatory-compliant systems in the future to enable interventional research that changes medical practice.

The concepts presented in this manuscript are independent of the implementation in a German hospital. For example, Garza et al. have shown examples of prototypes for eSource-based trial data from European countries, the United States of America and beyond^31,32^. These examples further illustrate the need for next-generation study databases.

Our study has several limitations: First, our proof of concept study had a prototypic character and scalability for multi-centric trials was not assessed. We conducted our research in conjunction with a single EHR system and feasibility of developing a similar workflow in other EHR systems needs further investigation. Further, we limited our study to one university hospital and feasibility to extend our approach to other institutions still requires testing.

In conclusion, next-generation study databases should be FAIR, regulatory compliant, EHR-integrated, and scalable EDC systems. Medical documentation and patient generated data should be included. EDC should support clinical decision support. We demonstrated in a proof of concept study that such systems are technically and clinically feasible.

## Methods

### Requirements of next-generation study databases for patient data

We identified desiderata for a more efficient and effective study database from ongoing study projects at Heidelberg University Hospital, Germany. Between May 2021 and July 2023, we gathered and analyzed 110 features and functionalities of electronic data capture (EDC) systems requested from the local EDC development team by data scientists and clinicians representing 11 clinical disciplines. For the process of collecting, assessing, and ordering these items, we used the *issues* function of our local GitLab-system (https://gitlab.com). Managing the software development process with this kind of system is considered best-practice since requirements can be directly linked to feature development of the EDC software. In monthly meetings, the EDC development team analyzed and summarized the requirements for improved study databases, as detailed in the results section.

### Technical setting for next-generation study databases in Heidelberg

After defining the requirements of a next-generation study database, the next logical questions were “Is implementation of a next-generation study database with these characteristics feasible in a real university hospital setting? If so, what limitations exist presently?” To answer these questions, we conducted a proof of concept study at Heidelberg University Hospital. The main EHR system at this hospital is i.s.h.med (https://www.cerner.com/de/de/loesungen/ishmed). We used the MDM-Portal as our FAIR infrastructure for Case Report Forms (CRFs). We selected OpenEDC^14^ as the EDC system for the case study because it is open-source (permitting custom extensions and interfaces) and CDISC ODM-compliant. We installed OpenEDC as client-server system in the hospital’s intranet. We captured patient data (using iPad tablet computers) and medical documentation by healthcare professionals (using personal computers) in OpenEDC. We created patient-specific hyperlinks in the EHR system to the web-based graphical user interface of OpenEDC (frontend integration). For data transfer from EDC to EHR, we generated files in the Portable Document Format (PDF) and sent them to the EHR system with a Health Level 7 (HL7, https://www.hl7.org) version 2-based communication server (Orchestra version 4.10 from x-tention Informationstechnologie GmbH, https://x-tention.com/en/overview/orchestra-ehealth-suite). The Medical Data Integration Center (MeDIC) of Heidelberg University Hospital implemented a data exchange from the EHR to the EDC and provided custom ETL routes for selected clinical data elements. We developed a CDS prototype as a web service.

### Digital medical history proof of concept study with EHR integration

**Fig. 6:**
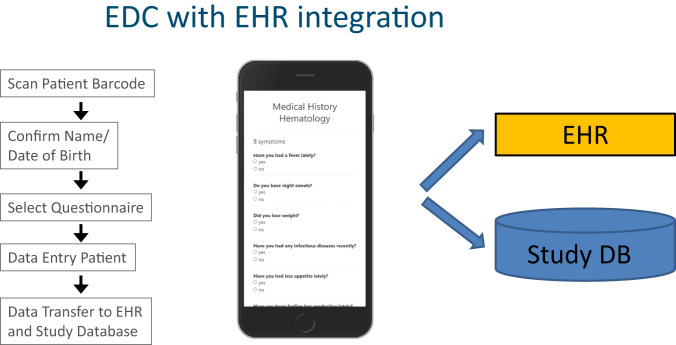
EDC workflow with EHR integration. Patient barcode is scanned with an iPad to identify the patient. After confirmation of patient’s name and date of birth, a suitable questionnaire is selected and the iPad is handed to the patient for data entry. When data collection is completed, data are transferred simultaneously into EHR system and into study database.

To assess technical and clinical feasibility of a next-generation study database, we conducted a proof of concept study on digital medical history with EHR integration at Heidelberg University Hospital. In the eleven-month pilot phase (September 2022 – July 2023) the data for 2,217 patients were documented in 19 EDC systems by 11 clinical departments (surgery, hematology, pediatrics, anesthesiology, radiation oncology, ophthalmology, gynecology, dermatology, gastroenterology, psychosomatics, and psychiatry) using the workflow of EDC with EHR integration as presented in Fig. 6. Patients answered the medical history questions on iPads provided for this task. Collected data were transferred simultaneously into the EHR system and into a study database (CDISC ODM format).

### Supplementary information

Supplemental Material

## Data Availability

This study is not based on EHR-records. Instead, the basis for requirements elicitation were issues from the local GitLab systems of Heidelberg University Hospital, since these issues contain confidential information they cannot be made available outside the institution.

## Code availability

The code of a containerized version of OpenEDC is available at https://github.com/OpenEDC/OpenEDC-Docker along with a tutorial how to run it. On https://medical-data-models.org/ a standalone version of OpenEDC can be run directly in a web browser after registration (free of charge).
