# Supplementary material for: Next-generation study databases require FAIR, EHR-integrated, and scalable Electronic Data Capture for medical documentation and decision support
Source: NPJ Digit Med. 2024 Jan 12;7:10. doi: 10.1038/s41746-023-00994-6 (PMC10786912; doi:10.1038/s41746-023-00994-6)
Supplement: Supplementary file 1 — Supplemental Material [file 41746_2023_994_MOESM1_ESM.pdf]

Supplementary Information

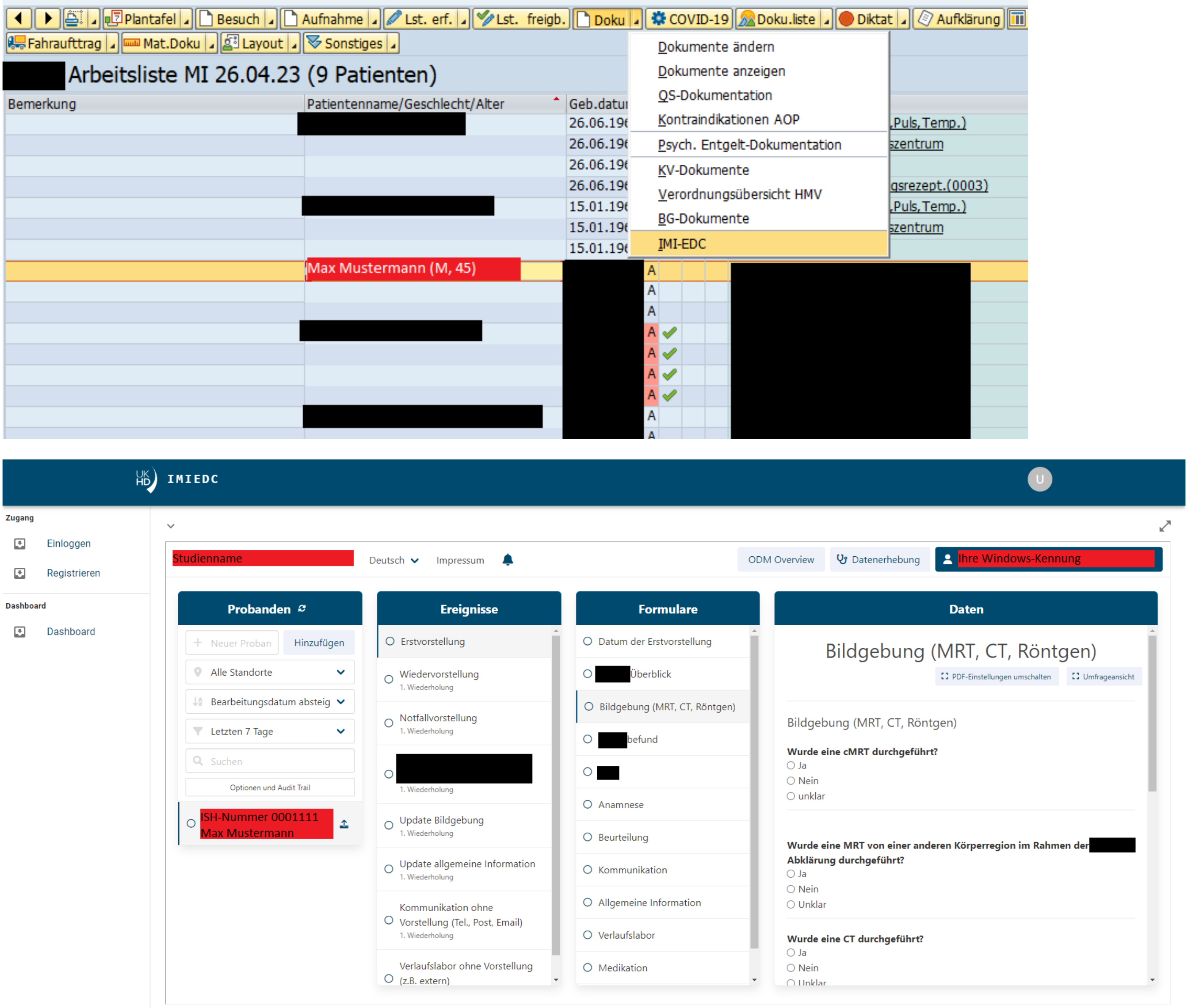

Supplementary Figure 1: Integration of EDC into clinical EHR workstations: A patient work list ("Arbeitsliste") in the legacy EHR system (upper panel) provides a web-link ("IMI-EDC") to the study database (lower panel), where data related to medical imaging ("Bildgebung") is captured. This web-link provides a single-sign-on functionality maintaining patient and user context and is available at any clinical workstation. Note: No actual patient data are presented here.
